# Research on equity of medical resource allocation in Yangtze River Economic Belt under healthy China strategy

**DOI:** 10.3389/fpubh.2023.1175276

**Published:** 2023-06-26

**Authors:** Liu Ya-qing, Niu Hao-ran, Tian Xiang-yang, Zhang Mei-cheng, Jiang Feng, Qian Yu-tong, Cao Jian-bo

**Affiliations:** ^1^School of Medicine and Health Management, Tongji Medical College, Huazhong University of Science and Technology, Wuhan, Hubei, China; ^2^China State Construction Northwestern Regional Headquarters, China State Construction Silkroad Construction Investment Group co., LTD, Xian, Shanxi, China; ^3^School of Marxism, Party School of CPC Hubei Provincial Committee, Wuhan, Hubei, China

**Keywords:** Yangtze River Economic Belt, data envelopment analysis, healthy China strategy, equality, health equalities

## Abstract

**Objective:**

This study aimed to assess the fairness of medical resource allocation in the Yangtze River Economic Belt, based on the Healthy China strategy. It aimed to identify the issues with resource allocation fairness and provide optimization suggestions.

**Methods:**

To assess the allocation fairness from a geographical population perspective, the study used the Health Resource Concentration and Entropy Weight TOPSIS methods. Additionally, the study analyzed the allocation fairness from an economic level angle, using the Concentration Curve and Concentration Index.

**Results:**

The study found that the downstream area had higher resource allocation fairness than the midstream and upstream areas. The middle reaches had more resources than the upper and lower reaches, based on population concentration. The Entropy Weight TOPSIS method found that Shanghai, Zhejiang, Chongqing, and Jiangsu had the highest comprehensive score index of agglomeration. Furthermore, from 2013 to 2019, the fairness of medical resource distribution gradually improved for different economic levels. Government health expenditure and medical beds were distributed more equitably, while general practitioners had the highest level of unfairness. However, except for medical and health institutions, traditional Chinese medicine institutions, and primary health institutions, other medical resources were mostly distributed to areas with better economic conditions.

**Conclusion:**

The study found that the fairness of medical resource allocation in the Yangtze River Economic Belt varied greatly based on geographical population distribution, with inadequate spatial accessibility and service accessibility. Although the fairness of distribution based on economic levels improved over time, medical resources were still concentrated in better economic areas. The study recommends improving regional coordinated development to enhance the fairness of medical resource allocation in the Yangtze River Economic Belt.

## 1. Introduction

The Yangtze River Economic Belt is one of the China’s three strategic regions (1), spanning 11 provinces (cities) in the east, central, and western regions. It covers approximately 21.4% of China’s total land area, and its population and GDP account for more than 40% of the country’s total. The middle and lower reaches of the Yangtze River Economic Belt are located in the central and eastern parts of China. The western region is dominated by agricultural economy and has abundant natural resources such as coal, oil, and natural gas, which have led to the development of heavy industry. The eastern region, on the other hand, is the center of economic activity, with a significant portion of exports coming from this area. The government has increased investment in infrastructure and education in the region, which has helped to promote economic growth. The Development Plan of the Yangtze River Economic Belt, issued in 2016, aims to promote the coordinated development of the upper, middle, and lower reaches of the Yangtze River (2).

The Healthy China plan emphasizes the need to “promote the coordinated development (3) of medical and healthcare in Beijing, Tianjin, Hebei, the Yangtze River Economic Belt, and other regions.” The 14th Five-Year Plan^1^ proposes comprehensive development (4) of the Yangtze River Economic Belt. With the implementation of the Healthy China Strategy and the 14th Five-Year Plan, the medical and health services in the Yangtze River Economic Belt have continued to develop. The scale of medical resource investment has grown rapidly, and the medical and health needs of residents have gradually been met, showing high growth, diversification, and multi-level characteristics (5). However, there are still problems (6) such as insufficient and unbalanced medical resource allocation and large regional distribution differences, resulting in contradictions between the supply and demand of medical resources. The fairness of medical resource allocation is directly related to equal and balanced satisfaction of medical and health needs and profoundly affects the fairness and stability of society.

The fairness of health services is an important indicator of the World Health Organization’s performance evaluation of health services, and the fairness of health resource allocation is the prerequisite for the fairness of health services (7). There have been numerous studies on health equity, and various methods have been employed to analyze the fairness of distribution of medical resources. These include the Gini coefficient and Lorentz curve, Theil index, and concentration index. For instance, Yu et al. (8) used the Lorentz curve and Gini coefficient to quantitatively analyze the fairness of distributing doctors in 31 provincial administrative regions of China, considering both population and geography. Chu et al. (9) proposed a method for allocating medical resources among hospitals during a public health emergency, using data envelopment analysis (DEA), and analyzed the fairness of distribution using the Lorentz curve and Gini coefficient.

Hyldgård (10) applied the concentration index to study stroke patients admitted to Danish public hospitals and found that income inequality was generally higher than educational inequality. Amir (11) used a concentration index to measure the socio-economic inequality of stillbirths in pregnant women in Tehran, Iran, and concluded that stillbirths are unevenly distributed among Iranian women, mainly in those with low economic status. Aghapour (12) used Theil index and mean logarithmic deviation to decompose the inequality within and between groups of self-financing health expenses in Iranian households.

From the perspective of research subjects, most studies on fairness are conducted at the regional or national level. Wang et al. (13) divided China into eight economic regions and analyzed the equity of primary healthcare, finding that equity in the northwest was significantly lower than in the southwest, while the coastal areas in the east had an excess of resources. Qiu et al. (14) evaluated the fairness and efficiency of health resource allocation in western China, quantitatively analyzing the distribution fairness from both population and geographic dimensions using Lorenz curves and Gini coefficients. The ratio of the concentration of medical and health resources to the concentration of population in 12 provinces and autonomous regions in western China ranged from 0.007 to 1.979, with Chongqing having the highest concentration ratios of 1.489, 1.979, 1.780, 1.879, and 1.851, while Tibet and Qinghai had ratios ranging from 0.007 to 0.054, indicating poor fairness in the allocation of health resources in western China. Li et al. (15) conducted a comprehensive assessment of the overall equity of Traditional Chinese Medicine (TCM) resource allocation in 2022 and found that the analysis of population-based TCM resource allocation equity was superior, but attention should be paid to rural areas with smaller populations. Given the vast territory of China and cultural and lifestyle differences among different provinces, analyzing the Yangtze River Basin rather than the entire country can reduce the influence of other factors.

Thus, it is essential to scientifically plan medical resources under the guidance of the Healthy China strategy, promote the improvement of the fairness of medical resource allocation, and better meet the medical and health needs of residents during the construction of the 14th Five-Year Plan in the Yangtze River Economic Belt. This paper uses the methods of health resource agglomeration degree and entropy weight TOPSIS method, concentration curve, and concentration index to explore the fairness of medical resource allocation in the Yangtze River Economic Belt from the perspective of geographical population and economic level, aiming to provide some reference for promoting the development of medical and health undertakings in the region.

## 2. Method

### 2.1. Data resources

**Figure 1:**
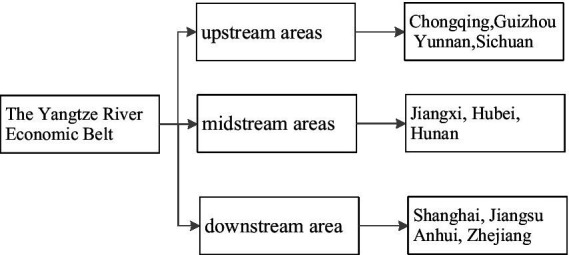
Eleven provinces (cities) along the Yangtze River Economic Belt.

In this study, all provinces (and cities) along the Yangtze River Economic Belt from 2013 to 2019 were selected as the research objects, as shown in Figure 1. The research data mainly came from the “China Statistical Yearbook,” “China Health and Health Statistics Yearbook,” and some from the health and health statistical yearbooks of various provinces (cities) in the Yangtze River Economic Belt.

### 2.2. Measuring tools

#### 2.2.1. Concentration of health resources

The concentration of health resources is an indicator that reflects the proportion of medical and health resources concentrated in a research area, accounting for 1% of the geographical area of the higher-level research area. The calculation formula (16) is as follows:


(1) $$HRAD_{i}=\frac{\left(\frac{HR_{i}}{HR_{n}}\right)\times 100\%}{\left(\frac{A_{i}}{A_{n}}\right)\times 100\%}=\frac{\frac{HR_{i}}{A_{i}}}{\frac{HR_{n}}{A_{n}}}$$

Population agglomeration is an indicator that reflects the population ratio of a research area to 1% of the geographic area of the next-level research area. The calculation formula (8) is:

(2) $$PAD_{i}=\frac{\left(\frac{P_{i}}{P_{n}}\right)\times 100\%}{\left(\frac{A_{i}}{A_{n}}\right)\times 100\%}=\frac{P_{i}/A_{i}}{P_{n}/A_{n}}$$

In the above calculation formula, $HRAD_{i}$ represents the concentration of health resources in a research area, $HR_{i}$ represents the number of certain types of medical resources owned by a research area *i*, and $HR_{n}$ represents the total amount of medical resources owned by the higher-level research area. $A_{i}$ represents the geographical area of a research area *i*, and $A_{n}$ represents the total geographical area of the higher-level research area. $PAD_{i}$ refers to the population concentration of a research area *i*, $P_{i}$ indicates the population of a research area *i*, and $P_{n}$ indicates the total population of a higher-level research area.

Compared to commonly used resource allocation fairness evaluation methods such as the Gini coefficient and differentiation index, the health resource agglomeration degree takes into account the geographical population factors of the Yangtze River Economic Belt (17). This paper examines the allocation fairness of population size through the health resource agglomeration degree and population agglomeration degree ratio.

#### 2.2.2. Entropy right TOPSIS method

The Entropy right TOPSIS method involves determining the entropy weight of various evaluation indicators using the entropy right method. It then calculates the comprehensive value of each evaluation index and employs the TOPSIS method for sorting and comparison (18). Essentially, the method normalizes different indicators in different dimensions. Compared to the traditional TOPSIS method, determining the weight of the indicator using the entropy method can produce a more objective result (19). The expression formula is as follows:

(3) $$C_{i}=\frac{D_{i}^{-}}{D_{i}^{+}+D_{i}^{-}}$$

$C_{i}$ is the composite score index (relative proximity), $D_{i}^{+}$ is the positive ideal solution distance, and $D_{i}^{-}$ is the negative ideal solution distance.

#### 2.2.3. Concentration curve and concentration index

The concentration curve measures the fairness of medical resource distribution across different economic levels. The degree of unfairness increases as the area between the concentration curve and the absolute fairness line (diagonal line) decreases (20). The concentration index is twice the area between the concentration curve and the absolute fairness line, with a range of values from −1 to 1. A larger absolute value indicates a greater degree of unfairness in the allocation of medical resources. The calculation formula is as follows:

(4) $$S=\frac{1}{2}\sum_{i=0}^{n-1}\left(Y_{i}+Y_{i+1}\right)\left(X_{i+1}-X_{i}\right)$$

(5) CI=2×(0.5−S)

where *CI* represents the concentration index, *S* represents the area between the concentration curve and the absolute fairness line (diagonal), *Y_i_* is the cumulative percentage of medical resources in the *i*-th study area, and *X_i_* represents the cumulative percentage of population in the *i*-th study area.

### 2.3. Index selection

Based on the theme of the Healthy China 2030 strategy of “jointly building and sharing a healthy country” (3), and considering the availability of indicator data, this study used committee decision to select indicators for studying the fairness of medical resource allocation. These indicators can comprehensively reflect the allocation of medical resources such as personnel, finance, and materials in the Yangtze River Economic Belt. This study identified the key indicators shown in Table 1 to measure and evaluate the fairness of medical resource allocation.

**Table 1:** Determination of equity indicators for medical resource allocation under healthy China.

Primary indicators	Secondary index	Abbreviations
A1 Medical Material Resources	B1 Number of Medical and Health Institutions (Number)	Health institutions
	B2 Number of TCM Institutions (Number)	Chinese medicine institutions
	B3 Number of Beds in Medical and Health Institutions (Number)	Medical beds
	B4 Number of Beds in Primary Medical and Health Institutions (Number)	Grassroots beds
A2 Medical Human Resources	Number of B5 Health Technicians (persons)	Health technicians
	B6 Number of Practicing (Assistant) Physicians (Person)	Practitioner
	B7 Number of Registered Nurses (Person)	Registered nurse
	B8 number of primary health technicians	primary health technicians
	B9 Number of General Practitioners (Person)	General practitioner
A3 Medical Financial Resources	B10 Government health expenditure (billion yuan)	Gov-health expenditure

### 2.4. Statistical analysis method

This study uses Microsoft Excel to sort out the data, and carries out the calculation of agglomeration degree, entropy weight TOPSIS method, concentration index and concentration curve drawing.

## 3. Result

### 3.1. Agglomeration analysis of medical resource allocation in Yangtze River Economic Belt

#### 3.1.1. Concentration of medical resources by geographical area

The results of the calculation for health resource agglomeration (*HRAD*) and population agglomeration (*PAD*) in the Yangtze River Economic Belt for the year 2019 are presented in Table 2. A value of *HRAD* greater than 1 indicates relatively concentrated allocation of medical resources by geographical area, whereas a value less than 1 indicates insufficient allocation. When *HRAD* equals 1, the allocation of medical resources by geographical area is considered absolutely fair.

**Table 2:** Health resource agglomeration and population agglomeration in the Yangtze River Economic Belt in 2019 (1).

*HRAD*	Sanitation institutions	Sort	Traditional Chinese medicine institutions	Sort	Medical treatment beds	Sort	grassroots beds	Sort	Health technicians	Sort	PAD
Shanghai	4.744	1	5.323	1	12.162	1	6.466	1	15.562	1	13.320
Jiangsu	1.823	2	1.877	4	2.648	2	2.487	2	2.978	2	2.736
Zhejiang	1.801	3	2.585	3	1.811	3	0.762	9	2.466	3	1.999
Anhui	1.016	8	0.783	7	1.307	5	1.271	6	1.246	5	1.583
Jiangxi	1.208	6	0.680	9	0.853	8	0.964	7	0.784	8	0.985
Hubei	1.018	7	0.697	8	1.132	7	1.355	5	1.071	7	1.099
Hunan	1.461	4	1.002	6	1.266	6	1.493	4	1.151	6	1.143
Chongqing	1.374	5	2.964	2	1.482	4	1.752	3	1.316	4	1.319
Sichuan	0.922	9	1.142	5	0.682	10	0.787	8	0.596	10	0.597
Guizhou	0.881	10	0.586	10	0.802	9	0.751	10	0.742	9	0.724
Yunnan	0.315	11	0.316	11	0.376	11	0.358	11	0.376	11	0.387
Downstream	1.547	1	1.708	1	2.042	1	1.573	1	2.366	1	2.253
Midstream	1.239	2	0.806	3	1.385	2	1.189	2	1.528	2	1.082
s	0.723	3	0.882	2	0.610	3	0.566	3	0.640	3	0.589

Health Resource agglomeration and population agglomeration in the Yangtze River Economic Belt in 2019 (2)											
*HRAD*	Practice physician	Sort	Registration nurse	Sort	primary health technicians	Sort	General practice Doctor	Sort	Gov-health expenditure	Sort	PAD
Shanghai	15.213	1	15.614	1	12.176	1	18.456	1	24.432	1	13.320
Jiangsu	3.203	2	2.907	2	3.076	2	5.470	2	2.577	2	2.736
Zhejiang	2.605	3	2.301	3	2.130	3	3.174	3	2.146	3	1.999
Anhui	1.277	5	1.245	5	1.217	5	1.274	4	1.378	4	1.583
Jiangxi	0.755	8	0.779	8	0.826	8	0.480	8	1.073	6	0.985
Hubei	1.057	7	1.104	7	1.094	7	0.808	7	0.935	7	1.099
Hunan	1.168	6	1.218	6	1.141	6	0.939	6	0.926	8	1.143
Chongqing	1.305	4	1.335	4	1.358	4	1.162	5	1.306	5	1.319
Sichuan	0.586	10	0.591	10	0.658	10	0.431	10	0.574	10	0.597
Guizhou	0.666	9	0.744	9	0.781	9	0.438	9	0.868	9	0.724
Yunnan	0.337	11	0.387	11	0.375	11	0.238	11	0.407	11	0.387
Downstream	2.478	1	2.298	1	2.224	1	3.364	1	2.369	1	2.253
Midstream	1.010	2	1.051	2	1.033	2	0.761	2	0.972	2	1.082
Upstream	0.556	3	0.590	3	0.620	3	0.412	3	0.607	3	0.589

According to the results in Table 2, health resources in Shanghai, Jiangsu, and Zhejiang are highly concentrated with an HRAD greater than 1, ranking them among the top 3 in the economic belt. On the other hand, Sichuan, Guizhou, and Yunnan have an HRAD less than 1, indicating that the allocation of medical resources by geographical area is less fair and the concentration of health resources is relatively low. The downstream area has the highest agglomeration degree, with indicators, except for health institutions and primary health institutions, having a value greater than 2. This suggests that medical resources in the downstream area are overly concentrated in terms of geographical allocation. In the middle reaches of the region, the concentration of other indicators, except for traditional Chinese medicine institutions, ranks second. Moreover, except for general practitioners and government health expenditures, the concentration of other indicators is less than 1, indicating a relatively fair geographical allocation. Finally, the upstream area has a concentration value less than 1 for all indicators, indicating poor accessibility of medical resources and a lack of geographical allocation.

#### 3.1.2. Concentration of medical resources by population

Through the ratio of the concentration of health resources and the concentration of population, the fairness of the allocation of medical resources according to the population size can be analyzed. When the ratio is greater than 1, it means that the medical resources are surplus relative to the population size, and vice versa. When the ratio is close to 1, it means that the resources are allocated fairly according to the population size, and basically meet the medical needs of the concentrated population. See Table 3 for details.

**Table 3:** Ratio of health resource agglomeration to population agglomeration in the Yangtze River Economic Belt in 2019 (1).

*HRAD*	Sanitation institutions	Sort	Traditional Chinese medicine institutions	Sort	Medical treatment beds	Sort	Grassroots beds	Sort	Health technicians	Sort	PAD
Shanghai	0.356	11	0.400	11	0.913	8	0.485	10	1.168	2	13.320
Jiangsu	0.666	9	0.686	8	0.968	7	0.909	8	1.089	3	2.736
Zhejiang	0.901	7	1.293	3	0.906	9	0.381	11	1.234	1	1.999
Anhui	0.642	10	0.495	10	0.826	11	0.803	9	0.787	11	1.583
Jiangxi	1.226	3	0.691	7	0.867	10	0.979	6	0.796	10	0.985
Hubei	0.926	6	0.634	9	1.030	5	1.233	4	0.974	8	1.099
Hunan	1.278	2	0.877	4	1.108	3	1.306	3	1.007	5	1.143
Chongqing	1.042	5	2.247	1	1.123	2	1.328	1	0.998	7	1.319
Sichuan	1.545	1	1.914	2	1.142	1	1.319	2	0.998	6	0.597
Guizhou	1.216	4	0.810	6	1.107	4	1.037	5	1.025	4	0.724
Yunnan	0.814	8	0.815	5	0.972	6	0.924	7	0.970	9	0.387
Downstream	0.687	3	0.758	2	0.906	3	0.698	3	1.050	3	2.253
Midstream	1.145	2	0.745	3	1.280	1	1.099	1	1.412	1	1.082
Upstream	1.229	1	1.498	1	1.036	2	0.962	2	1.087	2	0.589

The ratio of health resource agglomeration to population agglomeration in the Yangtze River Economic Belt in 2019 (2)											
*HRAD*	Practice physician	Sort	Registration nurse	Sort	primary health technicians	Sort	General practice doctor	Sort	Gov-health expenditure	Sort	PAD
Shanghai	1.142	3	1.172	1	0.914	9	1.386	3	1.834	1	13.320
Jiangsu	1.171	2	1.063	4	1.125	1	2.000	1	0.942	8	2.736
Zhejiang	1.303	1	1.151	2	1.066	4	1.588	2	1.073	4	1.999
Anhui	0.807	10	0.787	11	0.769	11	0.805	6	0.871	9	1.583
Jiangxi	0.767	11	0.791	10	0.838	10	0.487	11	1.090	3	0.985
Hubei	0.962	7	1.004	7	0.995	7	0.735	7	0.850	10	1.099
Hunan	1.022	4	1.065	3	0.999	6	0.821	5	0.810	11	1.143
Chongqing	0.989	5	1.012	6	1.030	5	0.881	4	0.990	6	1.319
Sichuan	0.982	6	0.990	9	1.102	2	0.722	8	0.962	7	0.597
Guizhou	0.920	8	1.027	5	1.079	3	0.605	10	1.199	2	0.724
Yunnan	0.871	9	1.000	8	0.969	8	0.615	9	1.050	5	0.387
Downstream	1.100	1	1.020	1	0.987	2	1.493	1	1.051	1	2.253
Midstream	0.933	3	0.972	3	0.955	3	0.703	2	0.898	3	1.082
Upstream	0.945	2	1.002	2	1.054	1	0.700	3	1.031	2	0.589

According to Table 3, Sichuan, Hunan, Jiangxi, and Guizhou have relatively higher concentrations of health institutions allocated based on population size, indicating that the allocation of resources is sufficient. Conversely, Shanghai, Jiangsu, and Anhui rank in the bottom three in terms of this ratio, indicating that the allocation of health institutions is insufficient relative to the population size. Chongqing’s ratio is close to 1, indicating a fair allocation. The ratio of the middle and upper reaches of the Yangtze River is greater than 1, while the ratio of the lower reaches is much lower than 1, indicating the need to optimize and improve these indicators step by step in combination with the geographical population situation.

Regarding the allocation of traditional Chinese medicine institutions, Sichuan and Chongqing have the highest concentration, indicating relatively concentrated resources. Shanghai and Anhui rank the bottom two in terms of the ratio, indicating that the allocation of traditional Chinese medicine institutions is insufficient according to the population size. The ratio of traditional Chinese medicine institutions in the upper reaches is greater than 1, while the ratio in the middle and lower reaches is less than 1.

In terms of bed allocation based on population size, Sichuan, Chongqing, and Hunan have higher concentration levels. The ratio of the upper and middle reaches is greater than 1, while the ratio of the lower reaches is less than 1, indicating the need for further optimization in the allocation.

Regarding the allocation of beds in primary health institutions, Sichuan, Hunan, Hubei, and Chongqing have the highest concentration. The distribution of grassroots beds is relatively fair according to the population. The ratio of Shanghai and Zhejiang ranks second, indicating an insufficient allocation of grassroots beds according to the population size. The ratio of grassroots beds in the middle reaches is greater than 1, while the ratio in the lower reaches is much lower than 1.

Regarding the allocation of medical human resources by population size, Jiangsu and the lower reaches of the Yangtze River have a higher concentration of various indicators. In terms of the allocation of health technical personnel, Zhejiang has the most sufficient ratio relative to the population size, while Jiangxi and Anhui rank second. Health technical personnel are insufficiently allocated according to the population size, and Jiangsu’s ratio is greater than and close to 1, making it the fairest. The ratio of the upper, middle, and lower reaches is greater than 1, and the overall allocation in the Yangtze River Economic Belt is relatively fair.

In terms of the allocation of medical practitioners, Shanghai, Zhejiang, and Jiangsu have relatively sufficient allocations according to the population size, while Jiangxi and Anhui have insufficient allocations, resulting in poor fairness. The ratio of the lower reaches of the Yangtze River is greater than 1, while the middle and upper reaches are less than 1. Therefore, the allocation of medical practitioners still needs improvement.

Regarding the allocation of registered nurses, Shanghai and Zhejiang have a higher concentration relative to the population size. Jiangxi and Anhui rank at the bottom in terms of the ratio, indicating a relative lack of registered nurses. The ratios in Hunan and Jiangsu are greater than and close to 1, making them fairer. The ratio of upstream and downstream areas is greater than 1, while the ratio of the middle reaches is less than 1.

Concerning the allocation of primary health technicians, Sichuan and Jiangsu have relatively sufficient allocations according to the population size, while Jiangxi and Anhui rank at the bottom with poor concentration levels. The ratio of the upstream areas is greater than 1, while that of the middle and lower reaches is less than 1.

In the allocation of general practitioners, Shanghai, Zhejiang, and Jiangsu are more concentrated or even have a surplus relative to the population size. Jiangxi, Yunnan, and Guizhou rank at the bottom three, indicating a serious shortage relative to the population size. The ratios in other provinces and cities are less than 1. Additionally, the ratio of the lower reaches of the Yangtze River is greater than 1, while the middle and upper reaches are less than 1, indicating a need for further balance and strengthening of the allocation of general practitioners.

Regarding the concentration of government health expenditure in medical financial resources, Shanghai has the most concentrated government health expenditure relative to the population size, while Hubei, Hunan, and Anhui rank at the bottom three. The allocation of government health expenditure is less fair according to population size. The ratio of the upper and lower reaches of the Yangtze River is greater than 1, while that of the middle reaches is less than 1, indicating a need for improved allocation of government health expenditure in the middle reaches.

Overall, the medical resources in the provinces and cities of the Yangtze River Economic Belt are fair or poor according to the population size, with large differences between regions. Areas with high levels of economic development such as Shanghai and Zhejiang have relatively lacking primary medical resources, while areas with poor economic development such as Anhui and Jiangxi have insufficient high-level medical human resources such as general practitioners and practicing physicians.

#### 3.1.3. Analysis of agglomeration under the entropy right TOPSIS method

According to Table 4, from the analysis of the entropy weight method, the entropy weights of government health expenditures, traditional Chinese medicine institutions, and general practitioners in the provincial areas of the Yangtze River Economic Belt are the top 3, all located between 0.1 and 0.2. The level of medical resource agglomeration in the provincial region has a greater impact, and the entropy weight of grassroots health technicians is the lowest, which has the least impact on the level of medical resource agglomeration in the provincial region. Among the entropy weights in the upper, middle, and lower reaches, general practitioners, Chinese medicine institutions, and practicing physicians have the highest entropy weights, which have a greater impact on the overall concentration of medical resources, and the entropy weights of health institutions are the lowest, which has the smallest impact on the overall concentration level.

**Table 4:** Entropy and weight of medical resources in Yangtze River Economic Belt.

Region	Item	Sanitation institutions	Traditional Chinese medicine institutions	Medical treatment beds	Grassroots beds	Health technicians	Practice physician	Registration nurse	Primary health technicians	General practice Doctor	Gov-health expenditure
Provincial region	Information entropy value e	0.924	0.824	0.9	0.921	0.902	0.891	0.909	0.932	0.826	0.81
	Information utility value d	0.076	0.176	0.1	0.079	0.098	0.109	0.091	0.068	0.174	0.19
	Weight	0.065	0.152	0.086	0.068	0.084	0.094	0.078	0.059	0.15	0.164
Upper, middle and lower reaches Area	Information entropy value e	0.629	0.081	0.522	0.613	0.283	0.222	0.617	0.514	0.028	0.631
	Information utility value d	0.371	0.919	0.478	0.387	0.717	0.778	0.383	0.486	0.972	0.369
	Weight	0.063	0.157	0.082	0.066	0.122	0.133	0.065	0.083	0.166	0.064

The TOPSIS method is used to calculate the comprehensive score index (relative proximity) C *_I_* of each province (city) and the optimal scheme. The higher the comprehensive score index, the higher the ranking, and the higher the concentration level of medical resource allocation. In Table 5, it can be seen that Shanghai, Zhejiang, Chongqing, and Jiangsu rank the top 4 in comprehensive scores, and the level of comprehensive medical resources agglomeration is relatively high, while Yunnan, Jiangxi, and Anhui rank the bottom 3 in comprehensive scores, and the level of comprehensive medical resources agglomeration is poor. The comprehensive score of the lower reaches of the Yangtze River ranks first, and the comprehensive score of the middle reaches ranks first. It can be combined with factors such as geographical area, population size, and index weight to further improve the level of comprehensive medical resource allocation and improve the fairness of medical resource allocation.

**Table 5:** The level and ranking of medical resources agglomeration in the Yangtze River Economic Belt in 2019.

Region	Positive ideal solution distance (D^+^)	Negative ideal solution distance (D^−^)	Composite score index (C *_I_*)	Sort
Shanghai	0.357333 88	0.441325 68	0.552 58	1
Jiangsu	0.402735 32	0.365039 35	0.475 45	4
Zhejiang	0.341637 9	0.382983 3	0.528 53	2
Anhui	0.569318 64	0.086096 98	0.131 36	11
Jiangxi	0.529053 81	0.159807 87	0.231 99	10
Hubei	0.493761 54	0.209719 95	0.298 12	8
Hunan	0.465649 82	0.270070 67	0.367 08	7
Chongqing	0.377227 52	0.370957 65	0.495 81	3
Sichuan	0.398934 28	0.355426 55	0.471 16	5
Guizhou	0.418647 62	0.275165 91	0.396 60	6
Yunnan	0.468666 56	0.187975 58	0.286 27	9
Downstream	0.679557 24	0.622862 67	0.478 23	1
Midstream	0.781446 44	0.515995 92	0.397 70	3
Upstream	0.648840 81	0.587149 94	0.475 04	2

### 3.2. Research on fairness of economic level distribution based on concentration curve

#### 3.2.1. Analysis of the concentration curve of medical resource allocation

The concentration curve reflects the fairness of the distribution of medical resources between regions with different economic levels. Since 2013, the Yangtze River Economic Belt has developed rapidly, and the *per capita* GDP has shown a continuous upward trend, a higher political and economic status and continuous growth. The development trend provides a relatively stable economic foundation for the layout and investment of medical resources in the Yangtze River Economic Belt.

**Figure 2:**
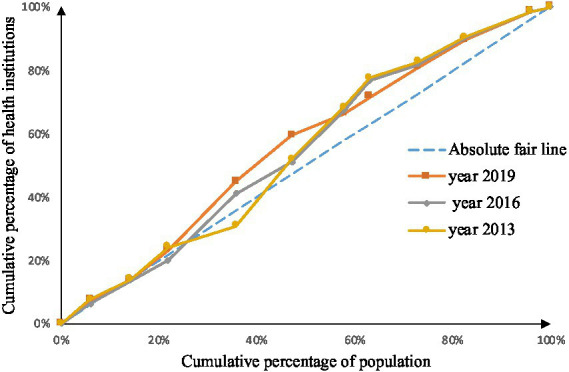
Concentration curve of medical and health institutions.

**Figure 3:**
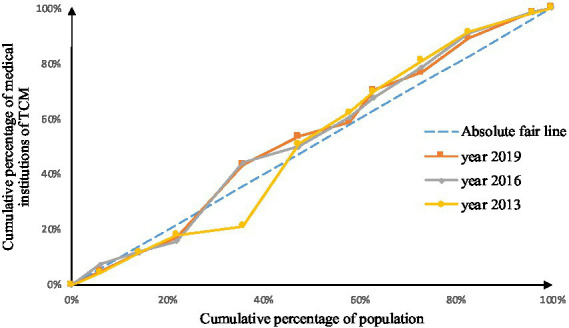
Concentration curve of traditional Chinese medicine institutions.

**Figure 4:**
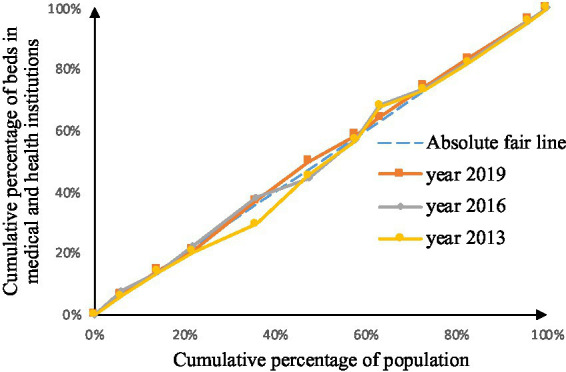
Concentration curve of beds in medical and health institutions.

**Figure 5:**
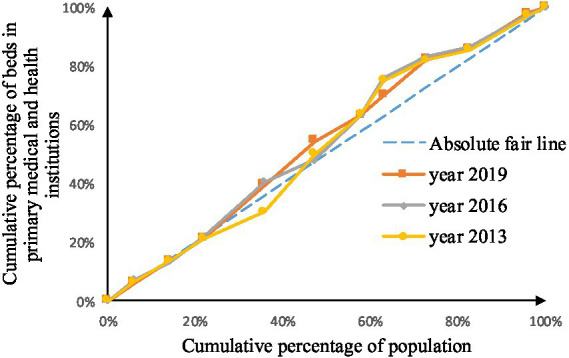
Concentration curve of beds in primary medical and health institutions.

**Figure 6:**
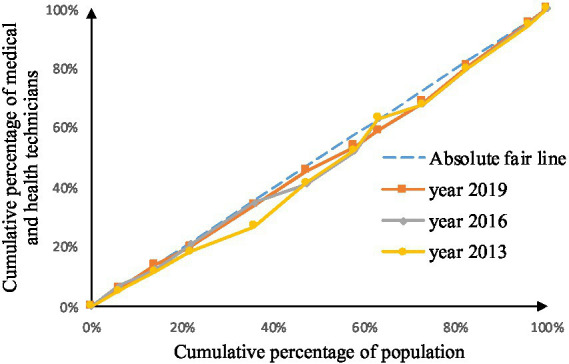
Concentration curve of health technicians.

**Figure 7:**
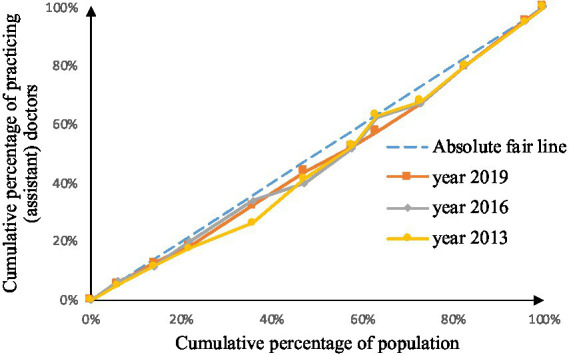
Concentration curve of practicing (assistant) physicians.

**Figure 8:**
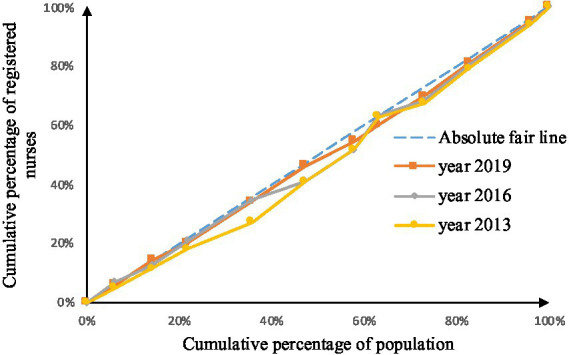
Concentration curve of registered nurses.

**Figure 9:**
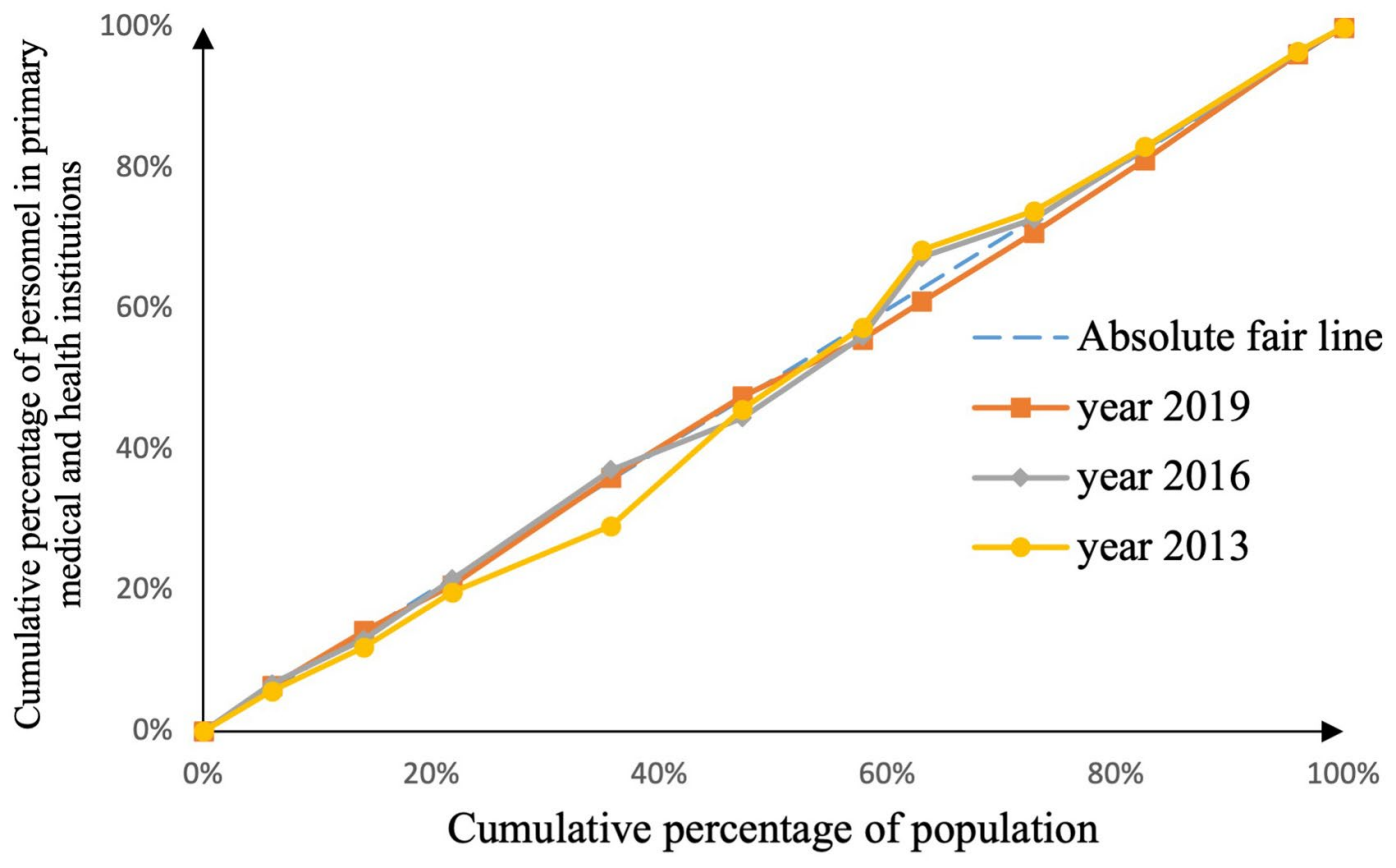
Concentration curve of primary health technicians.

As shown in Figures 2–9, the *per capita* GDP of the 11 provinces (cities) of the Yangtze River Economic Belt is arranged in ascending order, with the cumulative percentage of population as the horizontal axis and the cumulative percentage of medical resources as the vertical axis (21), and the concentrated curve of the distribution of medical resources in the Yangtze River Economic Belt is drawn. The concentration curve below the absolute fairness line indicates that resources are skewed toward better economic regions and vice versa.

Through Figures 2, 3, in the allocation of institutional resources, the concentration curve of health institutions is basically above the fair line, and the area between the fair line is larger, reflecting that health institutions in the Yangtze River Economic Belt are more inclined to be allocated in areas with poor economic levels. The concentration curve of Chinese medicine institutions is basically above the fair line, but the area between the fair line and the concentration curve of health institutions is smaller, indicating that although the allocation of Chinese medicine institutions in the Yangtze River Economic Belt also tends to be economic Level areas, but the fairness of distribution according to economic level is better.

Through Figures 4, 5, in the allocation of bed resources, the concentration curve of beds in medical and health institutions basically coincides with the fair line, and the area between the fair line is small, reflecting the different economic levels of the Yangtze River Economic Belt The distribution of bed resources in medical and health institutions is relatively fair. The bed concentration curve of primary medical and health institutions is basically above the fair line, and there is a certain area between the fair line, reflecting that the beds of primary medical and health institutions in the Yangtze River Economic Belt are relatively concentrated in areas with a low level of economic development.

After analyzing Figures 6–9, it is evident that the allocation of medical human resources in the Yangtze River Economic Belt favors areas with better economic conditions. The concentration curve for health technicians, practicing physicians, registered nurses, grassroots health technicians, and general practitioners all fall below the fair line, indicating a lack of fairness in resource allocation. The largest gap between the concentration curve and the fairness line is observed for general practitioners, followed by health technicians, practicing physicians, and registered nurses. Primary health technicians have the smallest gap, and their concentration curve is generally closer to the absolute fairness line. The allocation of general practitioners based on economic levels is the least fair, while the allocation of primary health technicians is relatively more equitable. However, the concentration curves of all five human resource indicators have gradually approached the fairness line from 2013 to 2019, suggesting an improvement in the fairness of resource allocation in the Yangtze River Economic Belt over time.

#### 3.2.2. Analysis of the concentration index of medical resource allocation

The value range of the concentration index of medical resources is [−1,1], the greater the absolute value, the greater the degree of unfairness, and the value of 0 is absolutely fair. According to Table 6, we can see the changes of medical resources in different economic levels in the Yangtze River Economic Belt during 2013–2019. Among them, general practitioners have the highest degree of unfairness, followed by health institutions, and government health expenditures, The absolute value of the concentration index of grassroots health technicians and medical beds is the closest to 0, and the fairness is the best. The fairness of the allocation of various medical resources according to the economic level is generally showing a trend of continuous improvement. In addition to health institutions, Chinese medicine institutions, and grassroots beds, the concentration of other medical resources is still inclined to areas with good economic conditions.

**Table 6:** The concentration index of medical resources in the Yangtze River Economic Belt, 2013–2019.

Year	Sanitation Institutions	Traditional Chinese Medicine Institutions	Medical treatment Beds	Grassroots Beds	Health technicians	Practitioner	Registered nurse	Primary health technicians	General practitioner	Government and health expenditure
2019	−0.119	−0.056	−0.018	−0.073	0.037	0.062	0.029	0.012	0.233	0.011
2018	−0.073	−0.012	0.004	−0.048	0.057	0.082	0.053	0.031	0.279	0.010
2017	−0.067	−0.008	0.008	−0.045	0.057	0.078	0.052	0.024	0.250	0.016
2016	−0.071	−0.031	0.008	−0.051	0.061	0.077	0.063	0.019	0.254	0.031
2015	−0.070	−0.002	0.013	−0.042	0.067	0.079	0.071	0.020	0.254	0.015
2014	−0.080	−0.008	0.010	−0.048	0.066	0.075	0.072	0.016	0.261	0.013
2013	−0.094	−0.022	0.012	−0.052	0.067	0.071	0.076	0.010	0.284	0.012

## 4. Discussion

### 4.1. The fairness of distribution by geographical population varies greatly, and the spatial accessibility and service accessibility are not in place

According to the allocation of medical resources in the Yangtze River Economic Belt by geographical area, the concentration of health resources is the highest in the lower reaches, particularly in provinces and cities such as Shanghai, Jiangsu, and Zhejiang, where the concentration of medical resources is too high. On the other hand, the middle reaches show a fair distribution of medical resources, while the upper reaches have the worst level of geographical concentration. Generally, there is a positive correlation between the level of economic development and the degree of geographical concentration, with economically better areas having a higher concentration of medical resources, and areas with poor economies having lower accessibility to medical resources (22). According to the allocation of medical resources by population size, there is significant variation in the distribution of medical resources across the region. The downstream areas have the best allocation of medical practitioners, general practitioners, and government health expenditures, whereas the middle and upstream areas show better equity in the allocation of medical and health institutions. The high population density in downstream areas makes it difficult to access medical material resources despite sufficient medical funds (23).

Using the entropy weight TOPSIS method, the highest entropy weight is assigned to government health expenditure, traditional Chinese medicine institutions, practicing doctors, and general practitioners, with health institutions having the smallest weight. The comprehensive score of the lower reaches of the Yangtze River ranks first, with Shanghai, Zhejiang, Chongqing, and Jiangsu having the highest concentration level of comprehensive medical resources, which is generally positively related to their economic level. The index with the highest entropy weight shows the importance of medical and health funds, traditional Chinese medicine undertakings, and high-level medical talents. Health institutions cannot represent the medical level only in terms of quantity (24), so they have the smallest weight. Improving the fairness of the allocation of medical resources in the Yangtze River Economic Belt can be considered from the perspectives of geographical area, population size, and high-weight indicators.

### 4.2. The fairness of the distribution of economic level continues to improve, but the overall tilt of resources toward better economic areas

The fairness of the allocation of medical resources in the Yangtze River Economic Belt based on different economic levels is showing a continuous improvement. Among them, the unfairness of the allocation of general practitioners, health institutions, practicing physicians, health technicians, and registered nurses is the highest, and government health expenditures, the fairness of grassroots health technicians and beds in health institutions is the best. Apart from healthcare institutions, traditional Chinese medicine institutions, and primary care beds, other medical resources are tilted toward economically better-off areas, which is similar to the findings of Xu et al. (25). Relevant research has shown that traditional Chinese medicine has significant advantages in medical services, prevention and health education in rural areas (26, 27). We speculate that the urbanization process has led to serious aging in rural areas, and the older adult have a higher acceptance of traditional Chinese medicine. It may also be due to the country’s strong development of primary healthcare and traditional Chinese medicine, which has led to a more widespread distribution of traditional Chinese medicine institutions and primary care beds. In any case, traditional Chinese medicine has great economic value in rural areas and plays an indispensable role in the development and stability of rural areas (28). Moreover, traditional Chinese medicine can complement the modern healthcare system and provide better healthcare services.

Among the indicators with the highest unfairness, except for health institutions that tend to be economically poor areas, other indicators that tilt to economically better areas are medical manpower indicators. This is because regions with better economic conditions have higher salaries and better career development opportunities. According to data from the China Statistical Yearbook 2022 (29), the healthcare industry income in eastern regions such as Shanghai and Jiangsu was 64.9 billion yuan and 95.56 billion yuan, respectively, while in western regions such as Shaanxi and Gansu, it was 32.04 billion yuan and 17.4 billion yuan, respectively. Similarly, a survey by Zhang et al. (30) found that the average salary for doctors in eastern regions was 139,736 yuan, higher than that in western regions at 105,060 yuan. The Matthew effect (17) applies to the supply of medical personnel, with economically developed regions attracting more medical human resources. The higher the level of medical talents, the stronger the flexibility and self-realization desire; therefore, general practitioners and practicing physicians are most inclined to economically developed areas. In recent years, Shanghai has issued the “Opinions on Reforming and Improving the Incentive Mechanism for the Training and Use of General Practitioners” and “About the City’s Reform and Improvement of the Incentive Mechanism for the Training and Use of General Practitioners.” The Implementation Opinions and enforcement of various medical talent introduction policies also confirm these conclusions to some extent. At the same time, high-quality resources are concentrated in areas with better economy, mostly large and medium-sized hospital institutions, with relatively limited (31) radiation scope and public health resources. Therefore, to improve the fairness of economic distribution, we need to pay attention to the construction of medical personnel in economically poor areas and the radiation range of medical institutions in economically better areas.

### 4.3. The Yangtze River Economic Belt should coordinate the coordinated development between regions and improve the fairness of medical resource allocation

China has a vast territory with complex and diverse terrain and an uneven distribution of population. Therefore, in future healthcare planning, factors such as service population, geographical heterogeneity, spatial accessibility, economic development level, healthcare market planning, and tailored equal planning should be comprehensively considered. The government should coordinate regional development, promote economic cooperation and industrial linkage advantages among the regions along the Yangtze River Economic Belt. The construction of the Belt and Road Economic Belt provides favorable experience for improving the fairness of healthcare resource allocation (32).

Furthermore, the government should take measures to promote equalization and comprehensively coordinate the development of basic medical services, strengthen resource investment and support for economically underdeveloped and sparsely populated areas, improve the rational distribution and vertical sinking of primary medical resources, alleviate the unreasonable phenomenon of over-concentration of resources in economically developed areas, improve the spatial accessibility of medical resources (31), increase support for underdeveloped and remote areas in the western region, and narrow the gap. It is also necessary to maintain and coordinate the development of the eastern, central, and western regions (33).

## 5. Conclusion

The geographical population distribution in the Yangtze River Economic Belt shows varying degrees of fairness, with issues surrounding spatial and service accessibility. Medical resources are overly concentrated in downstream areas such as Shanghai, Jiangsu, and Zhejiang, with inadequate funding and staffing in upstream regions. Despite improvements related to economic levels, overall resources still favor more prosperous areas. Notably, medical manpower indicators demonstrate a high level of unfairness toward economically stronger regions, with only health institutions being more prevalent in poorer areas. To address these imbalances, efforts should focus on coordinated development between regions in the Yangtze River Economic Belt to achieve greater fairness in the allocation of medical resources.

### 5.1. Limitations

Our indicators were determined through group discussions, and there may be some omissions that were not evaluated. Additionally, this study focused on analyzing the fairness of medical resource allocation from the perspectives of population and geography, but did not take into account the actual health status and healthcare needs of people in different regions. Furthermore, the study primarily analyzed quantitative indicators such as medical staff, facilities, and beds, without examining their actual service capacity.

## Data availability statement

The original contributions presented in the study are included in the article/supplementary material, further inquiries can be directed to the corresponding author.

## Author contributions

NH-r wrote most of the contents of the article. TX-y collected data and analyzed the data. LY-q designed and conceived the study. All authors provided several suggestions for manuscript, and contributed to the article.

## Funding

This work was supported by the Research and Planning Fund for Humanities and Social Sciences of the Ministry of Education (21YJA630062).

## Conflict of interest

TX-y was employed by China State Construction Silkroad Construction Investment Group co., LTD.

The remaining authors declare that the research was conducted in the absence of any commercial or financial relationships that could be construed as a potential conflict of interest.

## Publisher’s note

All claims expressed in this article are solely those of the authors and do not necessarily represent those of their affiliated organizations, or those of the publisher, the editors and the reviewers. Any product that may be evaluated in this article, or claim that may be made by its manufacturer, is not guaranteed or endorsed by the publisher.

## Footnotes

^1^China’s inaugural “Five Year Plan” was drafted in 1953 to outline the country’s economic development. These plans have since been used to guide China’s economic growth and reflect its laws of economic development. The healthcare 5 year plans are a part of the national 5 year plans and aim to achieve healthcare-related goals within the broader context of economic development.
